# The Expression of Activin Receptor-Like Kinase 1 (ACVRL1/ALK1) in Hippocampal Arterioles Declines During Progression of Alzheimer’s Disease

**DOI:** 10.1093/texcom/tgaa031

**Published:** 2020-07-28

**Authors:** Kelley E Anderson, Thomas A Bellio, Emily Aniskovich, Stephanie L Adams, Jan Krzysztof Blusztajn, Ivana Delalle

**Affiliations:** 1 Department of Pathology and Laboratory Medicine, Boston University School of Medicine, Boston, MA 02118, USA; 2 Department of Pathology and Laboratory Medicine, Lifespan Academic Medical Center, Warren Alpert Medical School of Brown University, Providence 02903 RI, USA

**Keywords:** ACVRL1, ALK1, hippocampus, immunohistochemistry

## Abstract

Cerebral amyloid angiopathy (CAA) in Alzheimer’s disease (AD)—deposition of beta amyloid (Aβ) within the walls of cerebral blood vessels—typically accompanies Aβ buildup in brain parenchyma and causes abnormalities in vessel structure and function. We recently demonstrated that the immunoreactivity of activin receptor-like kinase 1 (ALK1), the type I receptor for circulating BMP9/BMP10 (bone morphogenetic protein) signaling proteins, is reduced in advanced, but not early stages of AD in CA3 pyramidal neurons. Here we characterize vascular expression of ALK1 in the context of progressive AD pathology accompanied by amyloid angiopathy in postmortem hippocampi using immunohistochemical methods. Hippocampal arteriolar wall ALK1 signal intensity was 35% lower in AD patients (Braak and Braak Stages IV and V [BBIV-V]; clinical dementia rating [CDR1-2]) as compared with subjects with early AD pathologic changes but either cognitively intact or with minimal cognitive impairment (BBIII; CDR0-0.5). The intensity of Aβ signal in arteriolar walls was similar in all analyzed cases. These data suggest that, as demonstrated previously for specific neuronal populations, ALK1 expression in blood vessels is also vulnerable to the AD pathophysiologic process, perhaps related to CAA. However, cortical arterioles may remain responsive to the ALK1 ligands, such as BMP9 and BMP10 in early and moderate AD.

## Introduction

The pathophysiology of Alzheimer’s disease (AD) is characterized by progressive accumulation of beta-amyloid (Aβ) deposits in the brain. In the parenchyma, Aβ is present as diffuse amyloid or in the form of plaques. In addition, Aβ deposits in the walls of blood vessels—a process referred to as cerebral amyloid angiopathy (CAA) (Greenberg et al. 2020). In CAA, Aβ deposits are predominantly found in the periphery of arterioles (Weller et al. 1998). CAA is pathogenic, associated with microbleeds (Yates et al. 2014) and cognitive defects (Arvanitakis et al. 2011) and is presumably caused by abnormal vessel structure leading to increased risk of hemorrhage or reduced local blood supply (Greenberg et al. 2020). Indeed, imaging results indicate that vascular dysregulation and cerebral hypoperfusion are associated with increased risk of dementia and accelerated cognitive decline (Iturria-Medina et al. 2016; Wolters et al. 2017). Therefore, it is important to understand the pathogenesis of vascular dysfunction in AD and thus, preserving vascular function is a therapeutic target for this disease. A key regulator of vascular development and function is the activin receptor-like kinase 1 (ALK1) transmembrane protein that acts as signaling receptor protein kinase for its circulating ligands BMP9/GDF2 and BMP10 (Brown et al. 2005; David et al. 2007; Scharpfenecker et al. 2007; Upton et al. 2009; Townson et al. 2012). ALK1 is broadly expressed in the endothelium (David et al. 2009; Pardali et al. 2010) where its activity is central for normal vascular development and remodeling (Roman and Hinck 2017). Mutations in the *ACVRL1* gene (reviewed in Abdalla and Letarte 2006), which encodes ALK1, cause hereditary hemorrhagic telangiectasia type II [OMIM #600376]—a disease characterized by arteriovenous malformations (Roman and Hinck 2017)—and are associated with pulmonary arterial hypertension (Trembath et al. 2001; Harrison et al. 2003; Yokokawa et al. 2020). We have previously reported that ALK1 protein is expressed in human and rat hippocampus and that its expression in human CA3 neurons is reduced in advanced, but not early stages of AD (Adams et al. 2018). Here we describe ALK1 expression in human hippocampal cortical and leptomeningeal blood vessels in autopsy brains in which AD pathology was accompanied by CAA. We show that ALK1 immunoreactivity in hippocampal arteriolar walls is reduced in AD patients, as compared with subjects with early AD pathologic changes that are either cognitively intact or with minimal cognitive impairment, irrespective of amyloid accumulation measured by the intensity of Aβ vascular immunohistochemistry (IHC) signal. Overall, the data indicate a similar pattern of neuronal and arteriolar loss of ALK1 in advancing AD and suggest that this loss may contribute to the mechanisms of vascular pathophysiology of AD, thus potentially targeting ALK1-agonist therapy (e.g., with BMP9/BMP10) in early stages of AD pathology as a strategy for improving vascular function in AD.

**Table 1:** Analyzed hippocampi of subjects organized according to Clinical Dementia Rating (CDR) score and Braak and Braak (BB) stage.

Subjects	BB stage; CERAD plaque density	Amyloid angiopathy	CDR score	Age	Sex	*APOE*	PMI (h)
Group I							
1	III; sparse	Mild	0	91	F	3/3	16.7
2	III; moderate	Severe	0	93	M	3/3	120
3	III; sparse	moderate	0.5	83	F	3/3	19.3
4	III; none	Severe	0.5	82	F	2/3	144
5	III; high	Severe	0.5	88	M	3/3	-
Group II							
1	IV; moderate	Moderate	1	89	M	3/4	3
2	IV; high	Moderate	1	92	F	-	7.4
3	V; high	Mild	2	83	M	3/4	3.5
4	V; high	Severe	2	90	F	3/3	24

Note: Cognitively intact subjects and subjects with mild cognitive impairment are in Group 1; AD patients are in Group 2.

## Materials and Methods

### Study Subjects and Human Postmortem Hippocampi

Human formalin-fixed paraffin-embedded (FFPE) tissue blocks of hippocampi were acquired through the Framingham Heart Study Brain Donation Program (Framingham, Massachusetts) and the Netherlands Brain Bank (Amsterdam, Netherlands) as described in Table 1. The study focused on arteriolar walls in hippocampal cortex and adjacent leptomeninges from individuals divided into 2 groups, matched for age and sex, based on clinical dementia rating (CDR) score (Blessed et al. 1968; Hachinski et al. 1975; Davis et al. 1991) and Braak and Braak (BB) stage (Braak and Braak 1991). The CDR was assigned based on antemortem assessment months before death and a postmortem retrospective CDR based on a family interview with one or more family members (Au et al. 2012). Group 1 included subjects either cognitively intact or with minimal cognitive impairment (CDR0-0.5) in the limbic BB stages (CDR0-0.5, BBIII; *n* = 5, age mean 87.4 years, 3 F/2 M), and Group 2 consisted of subjects with mild to moderate dementia (CDR1-2), definite AD by NINCDS-ADRDA criteria and the isocortical BB stages (CDR1-2, BBIV-V; *n* = 4, age mean 88.5 years, 2 F/2 M) (Table 1). All subjects had various degrees of CAA (mild to severe), similar distributions of vascular pathology (atherosclerosis, arteriolosclerosis, and infarcts) and the absence of non-AD neurodegenerative pathology with no Lewy body pathology reported in any of the subjects. The consortium to establish a registry for AD (CERAD) plaque density ranged from sparse to high in both groups. Only one subject in Group 1 had no neuritic plaques but did exhibit severe CAA. All subjects were de-identified, and authors were blinded to subjects’ CDR score and BB stage during data acquisition. Quantitative analysis of ALK1 immunoreactivity was conducted within the CA1 subregion distinctly identifiable at the level of the lateral geniculate nucleus.

### Antibodies

We analyzed arteriolar ALK1 using rabbit polyclonal anti-ALK1 antibody (1:25, HPA007041, Atlas Antibodies, Stockholm, Sweden) with previously characterized specificity (Adams et al. 2018). Mouse antihuman muscle specific actin (MSA; also known as alpha smooth muscle actin α-SMA) [HHF35] monoclonal antibody (0.22 μg/mL, Marque Corporation, Rocklin, CA) highlighted vascular walls in order to help select arterioles for analysis. Mouse antihuman amyloid-β (Aβ) [6F/3D] monoclonal antibody (1:25, Dako, Glostrup, Denmark) rabbit antihuman tau [A0024] polyclonal antibody (1:3200, Dako, Glostrup, Denmark), and mouse antihuman phospho-PHF-tau [AT8] monoclonal antibody (1:2000, Pierce, Rockford, IL) were also used for confirmation of neuropathological report data and qualitative analyses.

**Figure 1:**
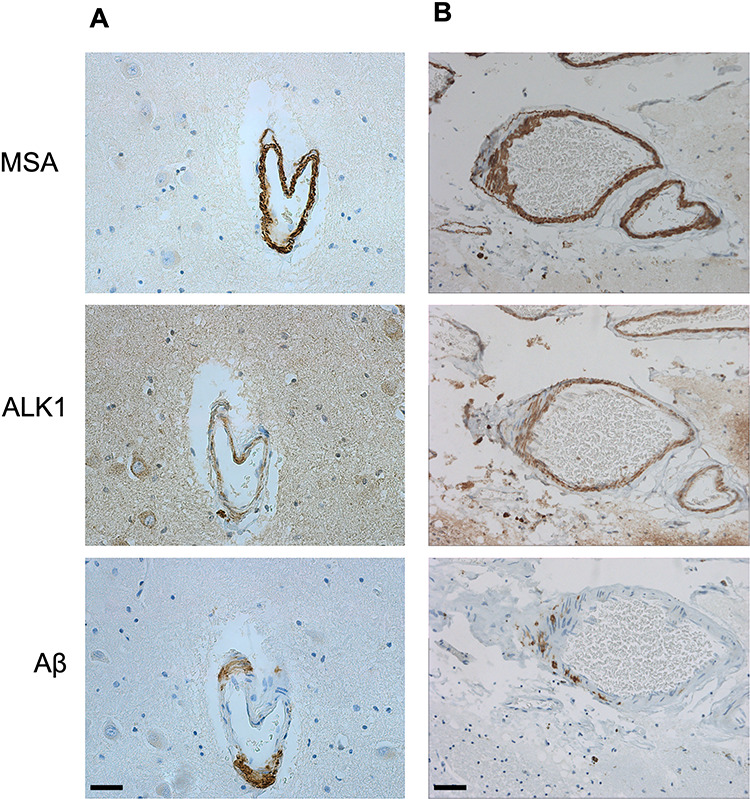
Identification of arteriolar walls selected for analysis. MSA signal was used to qualitatively identify and randomize arterioles in the hippocampal parenchyma (A, bar = 20 μm) and leptomeninges (B, bar = 40 μm). Once an arteriole was identified based on the presence of MSA in the vessel wall, the same arteriole was selected on both ALK1 and Aβ immuno-stained sections.

**Figure 2:**
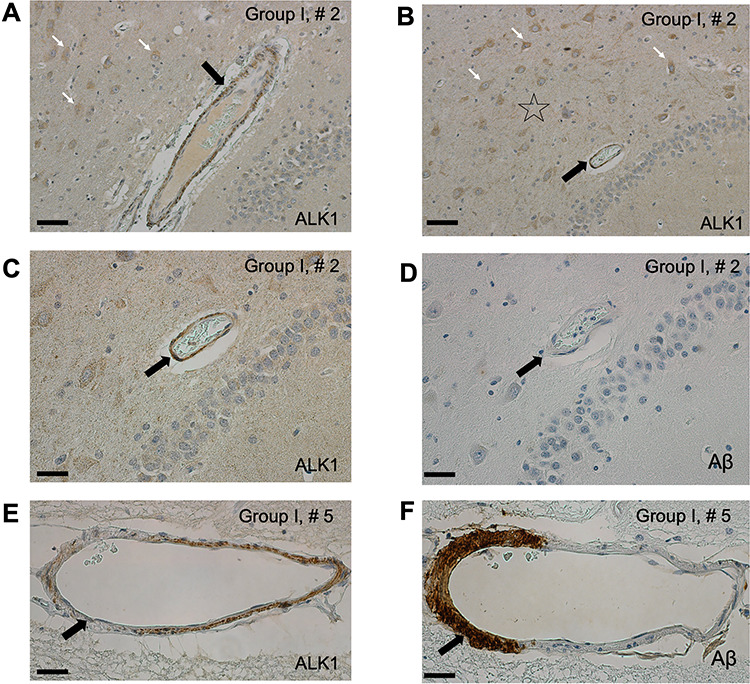
The arteriolar ALK1 signal is robust in hippocampal arterioles of non-AD subjects (Group I). The relatively strong ALK1 signal is present, not only in the cytoplasm of pyramidal neurons (white arrows) and in the neuropil (star), but also in the arteriolar walls (black arrow) in the hippocampal cortex and is representative of a pattern observed in non-AD individuals in Group I (*A*, *B*). ALK1 signal is uniformly strong in the arteriolar walls (*C*, arrow) without Aβ deposition (*D*, arrow). ALK1 signal appears faint (*E*, arrow) in the portion of the arteriolar wall with Aβ deposition (*F*, arrow) in a subject number 5 with a severe amyloid angiopathy in Group I. Bar = 40 μm (*A*, *B*); 20 μm (*C*–*F*).

### Immunohistochemistry

FFPE blocks were sectioned at 5 μm thickness, dried at room temperature for 24 h, and heated at 80 °C for 24 h before IHC processing. Deparaffinization, antigen retrieval, and subsequent staining was performed with Ventana Benchmark Ultra automated IHC instrument using Ventana Medical System reagents including ultraView Universal DAB (Cat#760-500), Hematoxylin II (Cat#790-2208), and Bluing Reagent (Cat#760-2037) (Ventana Medical Systems, Inc., Roche Diagnostics Ltd, Tucson, AZ) at the Boston Medical Center Pathology Department.

ALK1 protein and Aβ peptide expression was analyzed in 3 independent IHC experiments for each subject; in each experiment all the subjects were processed collectively. Therefore, experiments performed yielded 3 independently stained step-wise sections separated by at least 10 μm per subject for analysis. Automated IHC with the Ventana Benchmark Ultra allowed for maximally replicative conditions in IHC experiments, eliminating variability in reagent composition, quantity, incubation time, and human error, minimizing variability between experiments. Internal control sections from established subjects were stained collectively with any newly added subjects to ensure reproducibility of staining for the protein of interest. Quantitative analysis of ALK1 was generated from the imaged triplicate sections. Data from triplicate sections were averaged to obtain representative values for each subject.

### Quantitative Image Analysis

Slides were imaged using an Olympus BX60 light microscope, QImaging Retiga 2000R camera, and QCapture Suite and Suite PLUS software. For each subject, in order to capture (nearly) all the cortical and leptomeningeal arterioles identified on a single section, 10 ×40 cortical fields and 10 ×20 leptomeningeal fields of CA1-subiculum were imaged by 2 independent observers. Average immunoreactivity signals from 3 sections for each subject were obtained by automated IHC as previously described (Adams et al. 2016, 2017, 2018) (see above). Before the quantitative analyses of ALK1 and Aβ immunoreactive signals, MSA immunoreactivity was used to perform qualitative identification and randomization of arterioles in the hippocampal parenchyma and leptomeninges (Fig. 1). This approach also prevented bias that could arise from blood vessel selection based on the features of interest, that is, ALK1 and/or Aβ. All images used in quantitation were analyzed with ImageJ, version 1.8.0, Bethesda, MD: National Institutes of Health (Abramoff et al. 2004; Schneider et al. 2012). Intensity was quantified in ImageJ by converting the red, green, and blue (RGB) images to 8-bit grayscale images and subtracting background noise using a rolling bar radius. After outlining the blood vessel of interest, the image was inverted, and the lookup table was inverted. This created an image with inverted pixel values, with intensity values ranging from 0 (white) to 255 (black). Mean intensity values from the ×40 and ×20 field images in triplicate experiments comprised representative values for each subject.

### Data Presentation and Statistical Analyses

All individual data points are presented as well as means ±SEM. *P* value < 0.05 was considered statistically significant. The data were analyzed by *t*-test. Statistical analyses were performed with JMP software (Version 15.0.0 SAS Institute Inc., Cary, NC).

## Results

### ALK1 Protein Expression in the Hippocampal Parenchymal Arterioles Decreases in AD Irrespective of Amyloid Angiopathy

The relatively strong ALK1 signal is present not only in the cytoplasm of pyramidal neurons and neuropil in hippocampi of non-AD subjects (Adams et al. 2018) but also in the arteriolar walls (Fig. 2*A*–*C*). ALK1 signal is uniformly strong in the arteriolar walls free of Aβ deposition (Fig. 2*C*,*D*). We have occasionally observed faint or apparently absent ALK1 signal in the portions of the arteriolar walls bearing Aβ deposition (Fig. 2*E*,*F*; Supplementary Figure 1).

**Figure 3:**
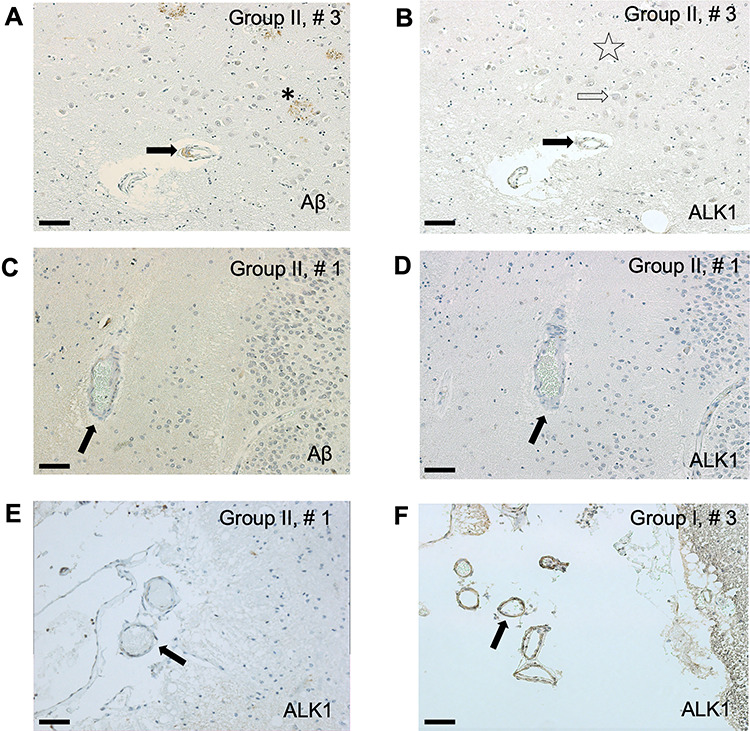
The arteriolar ALK1 signal appears reduced in hippocampal arterioles of AD subjects (Group II). As Aβ accumulates in the neuropil (neuritic plaques, star) and in the arteriolar walls (arrow) (*A*), ALK1 signal fades (*B*) in neuropil (star), cytoplasm of neurons (empty arrows) and vessel walls (black arrow). Even in the absence of amyloid angiopathy (*C*, arrow), ALK1 signal in the CA1 parenchymal arteriolar walls of an AD patient is faint (*D*, arrow). Similarly, in the same AD patient, ALK1 signal is severely reduced in the walls of leptomeningeal arterioles (*E*, arrow) in comparison with a non-AD subject (F, arrow). Bar = 40 μm (*A*, *B*, *E*, *F*); 20 μm (*C*, *D*).

**Figure 4:**
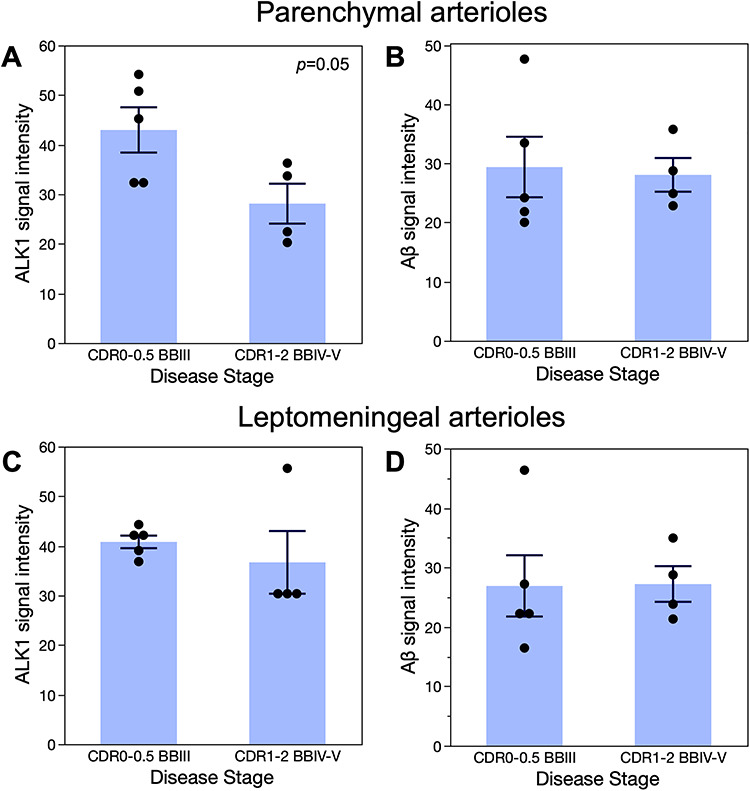
ALK1 immunoreactivity in hippocampal parenchymal arterioles declines in advanced AD. The intensity of the ALK1 (*A*, *C*) and Aβ (*B*, *D*) immunoreactivity in the parenchymal (*A*, *B*) and leptomeningeal (*C*, *D*) arterioles was determined as described in Methods. The original data points as well as means ± SEM are plotted on the graphs. The data were analyzed by *t*-test. There was a statistically significant decrease in ALK1 signal in advanced AD patients (CDR1-2; BBIV-VI) as compared with subjects with early AD-associated pathological changes (CDR0-0.5; BBIII). No other comparisons were statistically significant.

ALK1 signal in the parenchymal CA1 arteriolar walls decreased significantly in AD patients regardless of the presence of CAA in comparison with subjects with early AD neurofibrillary tangles accumulation (BB III) that were either cognitively intact (CDR0) or had mild cognitive impairment (CDR0.5) (Figs 3*A***–***D* and 4*A*,*B*). Although arteriolar walls in the hippocampal leptomeninges seem to exhibit weakened ALK1 immunoreactivity in AD patients (BBIV-V, CDR1–2) versus non-AD subjects (BBIII, CDR0-0.5) (Fig. 3*E*,*F*), quantitative comparison showed that only parenchymal and not leptomeningeal arteriolar walls undergo significant reduction in ALK1 signal in AD patients (Fig. 4*C*,*D*). That reduction is independent of the measured Aβ deposition signal associated with CAA.

## Discussion

In this study, we focused on the ALK1 expression in the hippocampal arteriolar walls in progressive stages of AD pathology. Our initial qualitative observations pointed to the possibility that the arteriolar wall regions with Aβ deposition were characterized by reductions, or even absence, of the ALK1 immunoreactive signal (Fig. 2*E*,*F*; Supplementary Figure 1), suggesting that CAA could lead to vascular ALK1 loss. Although this may be the case in individual vessels, our unbiased quantitative assessment of the vascular expression of ALK1 in the hippocampal parenchyma—in groups of subjects matched for age, sex, the degree of CAA, and vascular changes as well as the absence of non-AD pathology—indicates that the reduced ALK1 signal that accompanies AD progression is a feature of the arterioles in general (Fig. 4), apart from the presence of Aβ in the analyzed vessels or the global CAA evaluation of the subjects documented in the neuropathology reports.

The number of our subjects is, nevertheless, small. This is due to the criteria that we imposed at the onset of the study—that the subjects be matched for age and sex, that all have CAA, that none have non-AD pathology, and that they cover the CDR scores scale. *Apolipoprotein E4* allele (*APOE4*) allele happened to be present in some AD subjects but not in any of non-AD subjects. Postmortem interval varied greatly (Table 1) but obviously did not affect the quality of tissue processing or the protein expression. Again, our approach to IHC in human postmortem cortical sections (Adams et al. 2016, 2017, 2018) resulted in the reliable and reproducible yield of immunoreactivity signals in each subject. The range of ALK1 signal intensity did not exceed 16 and 23 units in cortical and leptomeningeal arteriolar walls, respectively, in any of the subjects. Similarly, the range of Aβ signal intensity did not exceed 22 and 13 units in cortical and leptomeningeal arteriolar walls, respectively, in any of the subjects.

The studies in living brains are still hampered by technological limitations when it comes to defining the relationship between Aβ deposition and the breakdown of the blood–brain. The breakdown of the blood–brain barrier has recently been suggested as a potential early biomarker for cognitive dysfunction in humans irrespective of positron emission tomography (PET)- and cerebrospinal fluid (CSF)-detected Aβ or tau accumulation (Nation et al. 2019; Montagne et al. 2020). The presence of one *APOE4* allele apparently promotes blood–brain barrier breakdown in cognitively intact (CDR0) and in mildly cognitively impaired (CDR0.5) individuals (Montagne et al. 2020).

Vascular Aβ deposits are thought to diminish blood flow and reduce vessel diameter potentially impeding Aβ clearance rate, promoting inflammation, and thus, likely contributing to neurodegeneration in AD (Koronyo et al. 2015; Bakker et al. 2016). Aβ accumulation in the muscular walls of cortical and leptomeningeal arterioles is similarly associated with the risk of large hemorrhage in the brain (Vonsattel et al. 1991). Vascular amyloidosis in the brains of AD patients and animal models is also accompanied by degeneration of pericytes leading to the altered permeability of the blood–brain barrier (Winkler et al. 2012; Halliday et al. 2016). Data from animal studies suggest intricate interplay between vascular Aβ accumulation (CAA), blood–brain barrier stability, and AD pathology. The mechanism underlying the association between CAA and cortical microhemorrhages (Vernooij et al. 2008; van Veluw et al. 2016) has been recently probed in APP/PS1 mice with CAA (van Veluw et al. 2020). Although the presence of vascular Aβ deposits in these mice did not directly predispose arterioles in their brains to leak, the physical alterations surrounding the vascular network likely contributed to the formation of spontaneous leakage sites (van Veluw et al. 2020). As in CAA, ALK1 deficiency in a genetic mouse model with focal cerebral *Alk1* gene inactivation was associated with compromised vascular integrity such as extravasation of intravascular components and reduced number of pericytes (Chen et al. 2013). Similarly, homozygous *Alk1* deletion in mice caused albumin extravasation in the retina (Akla et al. 2018). Moreover, in the same study, ALK1 expression was downregulated in the diabetic retinal blood vessels of wild type mice and *Alk1* heterozygotes (presumably expressing 50% of the wild type levels of the protein) were characterized by a dramatically exacerbated retinal vascular leakage evoked by diabetes, indicating *Alk1* haploinsufficiency. In the current study, we observed a 35% reduction in the apparent ALK1 levels in the arterioles of AD subjects, suggesting that the magnitude of this reduction could, by analogy with the mouse model, result in functional vascular defects. However, human studies on larger cohorts than ours are warranted.

Molecules at the point of convergence for neuronal and vascular pathology represent potentially doubly valid targets for a therapeutic intervention. We previously demonstrated that the immunoreactivity of ALK1 in CA3 pyramidal neurons is reduced in advanced, but not early stages of AD (Adams et al. 2018). Given that BMP9 administration ameliorates hippocampal AD-like pathology in mouse models of this illness (Burke et al. 2013; Wang et al. 2017), ALK1 may constitute a viable therapeutic target in early and moderate AD for the treatment of vascular abnormalities of this disease. Indeed, BMP9 administration ameliorated vascular diabetic retinopathy (Akla et al. 2018) and reduced pulmonary arterial hypertension in rat and mouse models by acting on endothelial cells (Long et al. 2015).

Our current data and published results (Adams et al. 2018) showing concomitant changes in vascular and neuronal ALK1 expression during AD progression are in line with our previous studies documenting simultaneous neuronal and arteriolar abnormalities in the expression of methionine sulfoxide reductase B3 (MSRB3) in hippocampi of AD patients (Adams et al. 2017). A single nucleotide polymorphism *rs61921502* in *MSRB3* is associated with the risk of low hippocampal volume and AD. We also investigated the relationship between the r*s61921502* G (minor/risk allele) and magnetic resonance imaging (MRI) measures of brain vascular injury and the incidence of stroke, dementia, and AD in 2038 Framingham Heart Study Offspring participants. When adjusted for age and age squared at MRI exam, sex, and *APOE4*), individuals with *MSRB3 rs61921502* minor allele and no *APOE4* had increased odds for brain infarcts on MRI (Conner et al. 2019).

Collectively, the data from our current and previous studies on ALK1 and MSRB3 (Adams et al. 2017, 2018; Conner et al. 2019) suggest that, in some cases, common molecular mechanisms may regulate vascular and neuronal function. These mechanisms may be vulnerable to pathophysiological processes, such as those of AD, in a similar fashion and thus be amenable to common therapeutic strategies. In the case of ALK1 dysfunction in early AD, these strategies could include treatment with agonists (Burke et al. 2013; Long et al. 2015; Wang et al. 2017; Akla et al. 2018) or with drugs that enhance ALK1-mediated signaling (Ruiz et al. 2017).

## Notes

We thank Terri Lima and Cheryl Spencer for expert IHC advice and assistance, Dr Joel Henderson for the use of imaging equipment, and Kerry Cormier of Framingham Heart Study Brain Bank and Michiel Kooreman of Netherlands Brain Bank for specimen procurement. *Conflict of Interest*: None declared.

## Funding

National Heart, Lung, and Blood Institute (contract no. N01-HC-25195, HHSN268201500001I). National Institutes of Health, National Institute on Aging (grants AG045031, AG057768).

## Supplementary Material

Supplemental_Figure_1_legend_tgaa031

Supplemental_Figure_1_tgaa031
