# Supplementary material for: The Expression of Activin Receptor-Like Kinase 1 (ACVRL1/ALK1) in Hippocampal Arterioles Declines During Progression of Alzheimer’s Disease
Source: Cereb Cortex Commun. 2020 Jul 28;1(1):tgaa031. doi: 10.1093/texcom/tgaa031 (PMC7497413; doi:10.1093/texcom/tgaa031)
Supplement: Supplemental_Figure_1_legend_tgaa031 [file supplemental_figure_1_legend_tgaa031.docx]

**Supplemental Figure 1. Apparently absent ALK1 signal in the portions of the leptomeningeal arteriolar walls bearing Aβ deposition in the hippocampus of a previously analyzed subject with amyloid angiopathy (Adams *et al.* 2018).** Aβ signal (**A**, asterisks) and ALK1 signal (**B**, asterisks) appear mutually exclusive. Insets ***c*** and ***d*** highlight the relationship between Aβ deposits (**C**, striped arrowheads) and ALK1-immunoreactivity (absent as indicated by striped arrowheads in **D**). ALK1 signal is present in arteriolar wall segments that are not involved by amyloid angiopathy (**D**, filled arrowheads). Scale bar = 25 μm.
