# Supplementary figures and images for: The Expression of Activin Receptor-Like Kinase 1 (ACVRL1/ALK1) in Hippocampal Arterioles Declines During Progression of Alzheimer’s Disease
Source: Cereb Cortex Commun. 2020 Jul 28;1(1):tgaa031. doi: 10.1093/texcom/tgaa031 (PMC7497413; doi:10.1093/texcom/tgaa031)

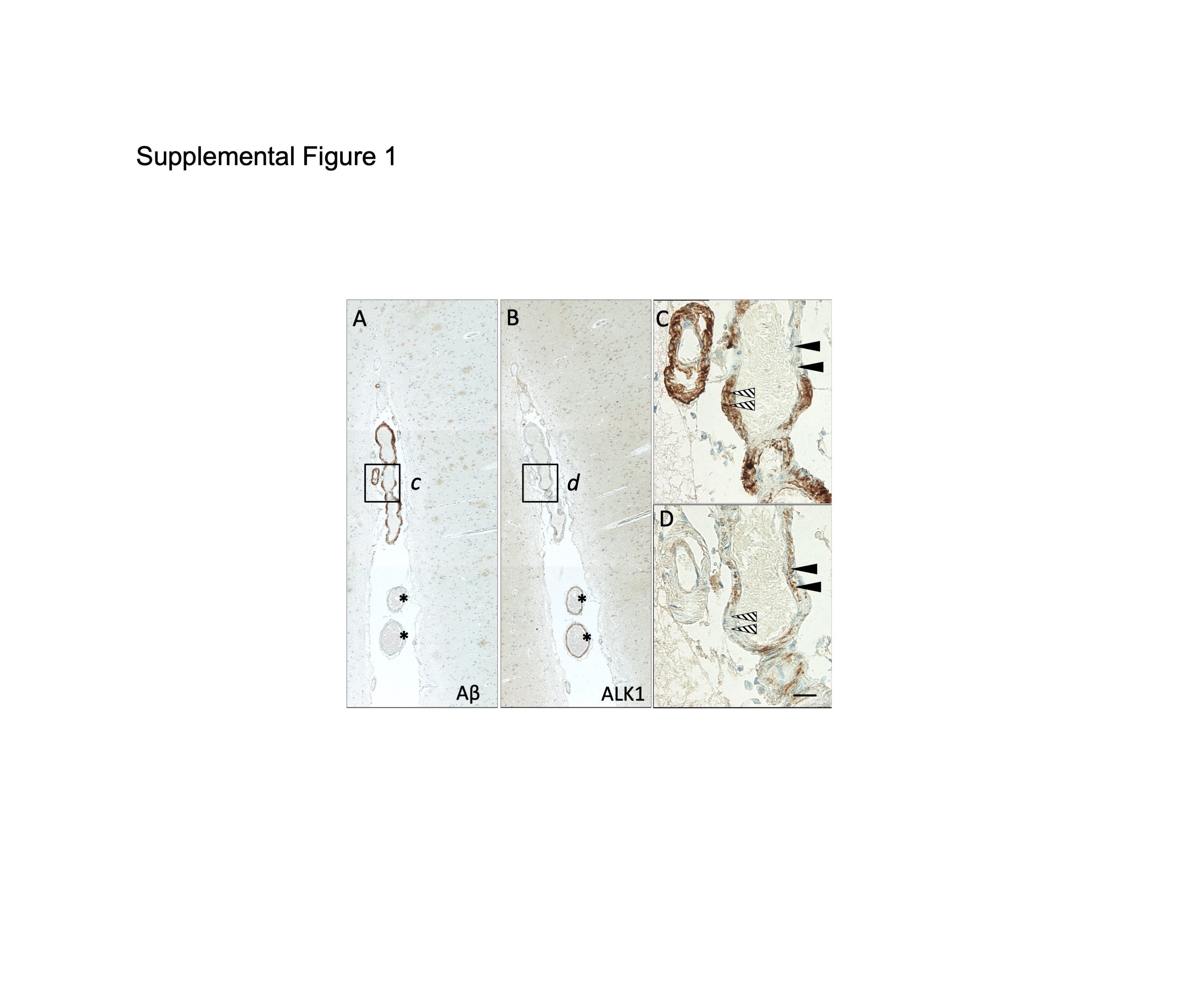

Supplement: Supplemental_Figure_1_tgaa031 [file supplemental_figure_1_tgaa031.jpeg]
